# Investigation of the Influence of Glucose Concentration on Cancer Cells by Using a Microfluidic Gradient Generator without the Induction of Large Shear Stress

**DOI:** 10.3390/mi7090155

**Published:** 2016-09-01

**Authors:** Tadashi Ishida, Takuya Shimamoto, Nobuya Ozaki, Satoshi Takaki, Takahiro Kuchimaru, Sinae Kizaka-Kondoh, Toru Omata

**Affiliations:** 1 Department of Mechano-Micro Engineering, Interdisciplinary Graduate School of Science and Engineering, Tokyo Institute of Technology, Kanagawa 226-8503, Japan; tokenai.shimamoto@gmail.com (T.S.); esorairam.1212@gmail.com (N.O.); smtwtfs25@gmail.com (S.T.); omata.t.aa@m.titech.ac.jp (T.O.); 2 Department of Biomolecular Engineering, Graduate School of Bioscience and Biotechnology, Tokyo Institute of Technology, Kanagawa 226-8501, Japan; tkuchimr@bio.titech.ac.jp (T.K.); skondoh@bio.titech.ac.jp (S.K.-K.)

**Keywords:** microfluidic gradient generator, perfusion culture, glucose concentration, cancer metastasis

## Abstract

A microfluidic device capable of precise chemical control is helpful to mimic tumor microenvironments in vitro, which are closely associated with malignant progression, including metastasis. Cancer cells under a concentration gradient of oxygen and other sustenance materials inside a tumor in vivo have recently been reported to increase the probability of metastasis. The influence of glucose concentration on cancer cells has not been measured well, whereas that of oxygen concentration has been thoroughly examined using microfluidic devices. This is because glucose concentrations can be controlled using microfluidic concentration gradient generators, which trade off temporal stability of the glucose concentration and shear stress on the cells; by contrast, oxygen concentration can be easily controlled without microfluidic device-induced shear stresses. To study cell division and migration responses as a function of glucose concentration, we developed a microfluidic device to observe cell behaviors under various chemical conditions. The device has small-cross-section microchannels for generating a concentration gradient and a large-cross-section chamber for cell culture. With this design, the device can achieve both a cell culture with sufficiently low shear stress on cell activity and a stable glucose concentration gradient. Experiments revealed that a low glucose concentration increased the total migration length of HeLa cells and that HeLa cells under a glucose concentration gradient exhibit random motion rather than chemotaxis.

## 1. Introduction

Understanding cancer cell metastasis is important because the survival rate of patients with metastasis is drastically lower than that of patients without metastasis in almost all cancers. The biochemical and genetic mechanisms of cancer metastasis have been widely studied both in vitro and in vivo [1,2,3]. In the initial step of the metastatic process, cancer cells gain higher migration capabilities [4,5] by losing adhesion to neighboring cells through epithelial-mesenchymal transitions which are induced by multiple mechanisms including a hypoxic environment [6,7,8,9,10]. Tumors have low oxygen concentrations owing to their abnormal structure and distribution of tumor blood vessels, through which sustenance such as oxygen and glucose are supplied [11]. Because of their high migration capability, cancer cells can reach and enter blood vessels, circulate throughout the body via the bloodstream, and migrate to other organs, resulting in metastasis [12]. The behavior of cancer cells under low oxygen concentrations has been actively studied; hypoxic cancer cells have been found to be epigenetically changed, contributing to their high migration capability [13] and induction of angiogenesis [14]. In addition to oxygen, glucose may also be a crucial factor for cancer cell metastasis [15,16,17], because glucose concentration is also low in chronic hypoxic regions in tumors. However, the influence of low glucose concentration on the metastatic activity of cancer cells is not fully understood.

To study the influence of glucose concentration on cancer metastasis, the mechanism at the cellular levels should be studied. To this end, in vitro approaches such as microscale visualization, reaction to chemical input, and gene manipulation are useful. However, results of in vitro studies sometimes correlate poorly with those of in vivo studies because the surrounding cellular environments are quite different. For example, sustenance concentration varies gradually over tumor regions. Considering that sustenance is exhausted at 2 × 10^2^ μm from the blood vessels [15,16] and that the blood glucose level of healthy adults is 50–180 mg/dL [18], the glucose concentration gradient is 1 × 10^−2^ to 5 × 10^−2^ mM/μm. In contrast, sustenance concentration around cancer cells in conventional in vitro experiments is uniform. This is because the experimental conditions can be controlled only through techniques such as pipetting chemical solutions into the culture dishes and gas exchange in incubators. Therefore, the culture conditions in vitro differ widely from those inside tumors. To accurately reproduce the behavior of cancer cells in tumors, the cancer cell culturing conditions must be similar to those in tumors. In this regard, microfluidic technologies are promising because they enable precise control over the conditions surrounding cancer cells [19,20,21]; the advantages of microfluidic devices are precise fluidic delivery, scalability, and cell manipulation [22,23]. Using these advantages of microfluidic devices, the influence of oxygen concentration on cancer cells has been precisely studied [19,24,25]. However, the influence of the glucose concentration on cancer cells has not been thoroughly studied. Unlike the oxygen concentration gradient, which can be controlled by adjusting the flow rate of oxygen and nitrogen gases, glucose concentration gradient can be controlled by adjusting the flow of high- and low-concentration glucose media. When the glucose media are directly introduced into a cell culture chamber, the glucose concentration gradient can be controlled by changing the flow rate of the glucose media, which in turn affects the shear stress on cells and thus changes the condition of the cancer cells. When the high- and low-concentration glucose media are separated using hydrogel walls and a cell culture chamber, a glucose concentration gradient is generated through diffusion without shear stress. However, spatially and temporally controlling the glucose concentration gradient [26] using this method is difficult.

To understand the influence of glucose concentration, we generated glucose concentration conditions that are similar to those inside a tumor by using a conventional network concentration gradient generator capable of direct introduction of high- and low-concentration glucose media. Using the concentration gradient generator, the temporal stability of the concentration gradient depends on the flow rate, while the flow rate affects cellular activities because of shear stress. In cellular activity such as division and migration, shear stress and change of the chemical composition should be kept stable and low, respectively. Therefore, a balance between the applied shear stress and the temporal stability of the concentration gradient is important. We achieved this balance between the applied shear stress and the temporal stability of the concentration gradient and observed the division and chemotaxis migration of cancer cells in solutions with uniform glucose concentrations and glucose concentration gradients.

## 2. Materials and Methods

### 2.1. Device

#### 2.1.1. Device Concept

**Figure 1:**
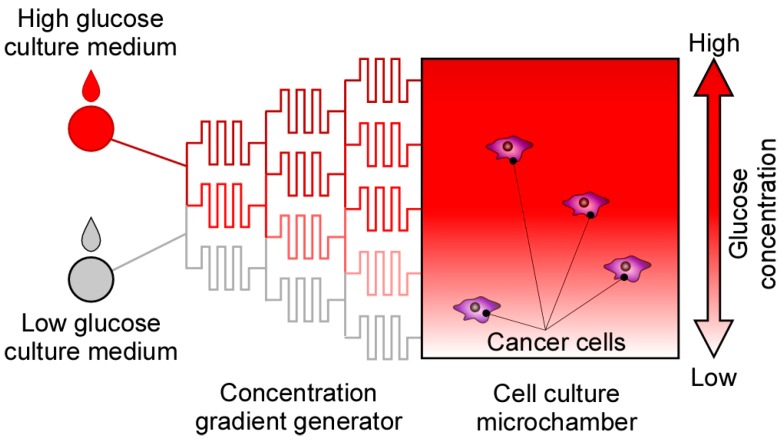
Schematic of the microfluidic device for investigating cell migration inside a tumor. High- and low-concentration glucose culture media were introduced into the concentration gradient generator, thus creating a glucose concentration gradient. Subsequently, behaviors of the cancer cells were observed inside the cell culture microchamber.

The microfluidic device is composed of two components: (1) a concentration gradient generator [27] and (2) a cell culture microchamber (Figure 1). The concentration gradient is generated using two culture media with high and low glucose concentrations. Media with different glucose concentrations are generated downstream and then flow into the cell culture microchamber. The following trade-off must be considered when using the microfluidic device: the concentration gradient temporally fluctuates because of trivial noises in the flow velocities of the input media. To neglect these fluctuations, media with high flow velocities should be used to ensure a temporarily stable gradient. However, such high-velocity media apply high shear stress on the cancer cells, resulting in poor culture conditions. Considering this trade-off, we designed a microfluidic device with small-cross-section microchannels for generating a glucose concentration gradient and a large-cross-section chamber that functions as the cell culture chamber.

#### 2.1.2. Fabrication Process

**Figure 2:**
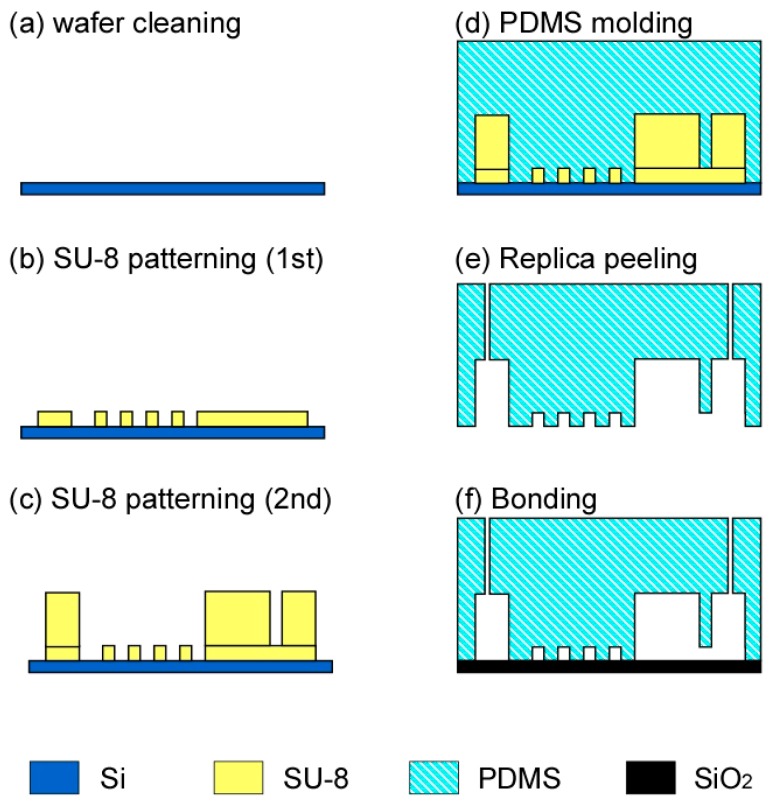
Fabrication of the microfluidic device. (**a**) Wafer cleaning with acetone and ethanol in an ultrasonic bath; (**b**) SU-8 patterning for the mold of the microchannels; (**c**) SU-8 patterning for the mold of the microchamber; (**d**) Polydimethylsiloxane (PDMS) curing; (**e**) Peeling off the PDMS replica from the mold; (**f**) Bonding the PDMS replica and a glass substrate.

The microfluidic device was fabricated through soft lithography [28] by using a microfabricated mold with two thicknesses [29], as depicted in Figure 2: (a) a silicon wafer was cleaned using acetone and ethanol in an ultrasonic bath for 10 min; (b) a 50-μm-thick SU-8 (SU-8 3050, Microchem, Westborough, MA, USA) was spin-coated and patterned on the wafer; (c) similarly, a 200-μm-thick SU-8 (SU-8 2150, Microchem, Westborough, MA, USA) was spin-coated and patterned on the same wafer. The SU-8 structure was exposed to CHF_3_ plasma to easily detach polydimethylsiloxane (PDMS, Silpot 184 W/C, Dow Corning, Midland, MI, USA) from the mold; (d) PDMS (a mixture of PDMS elastomer and cross-linker at a weight ratio of 10:1) was poured onto the mold and baked at 80 °C for 80 min. The microstructures of the mold were transferred to the PDMS replica; (e) the PDMS replica was peeled off; (f) the PDMS replica and a glass wafer were exposed to oxygen plasma for 10 s and bonded at 100 °C for 1 h.

#### 2.1.3. Temporal Stability of the Glucose Concentration Gradient

**Figure 3:**
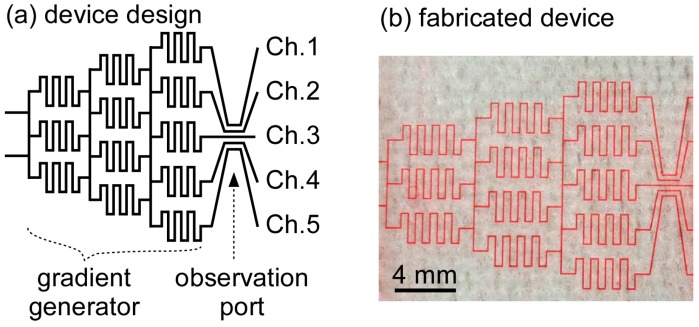
Microfluidic concentration gradient generator. (**a**) Device design. The gradient generator produces 5 different glucose solutions downstream. The microchannels are defined as Ch. 1–5; (**b**) Fabricated device filled with red-dyed solution.

The microfluidic concentration gradient generator is composed of symmetrically aligned microchannels with zigzag structures (Figure 3a). By ensuring a symmetric flow, media with high and low glucose concentrations generate media with high, average, and low glucose concentrations in the lower course of the microchannels. At the downstream microchannels, the aforementioned process is repeated, generating a glucose concentration gradient. Figure 3b shows the fabricated microfluidic concentration gradient generator (filled with red-dyed solution for visibility).

**Figure 4:**
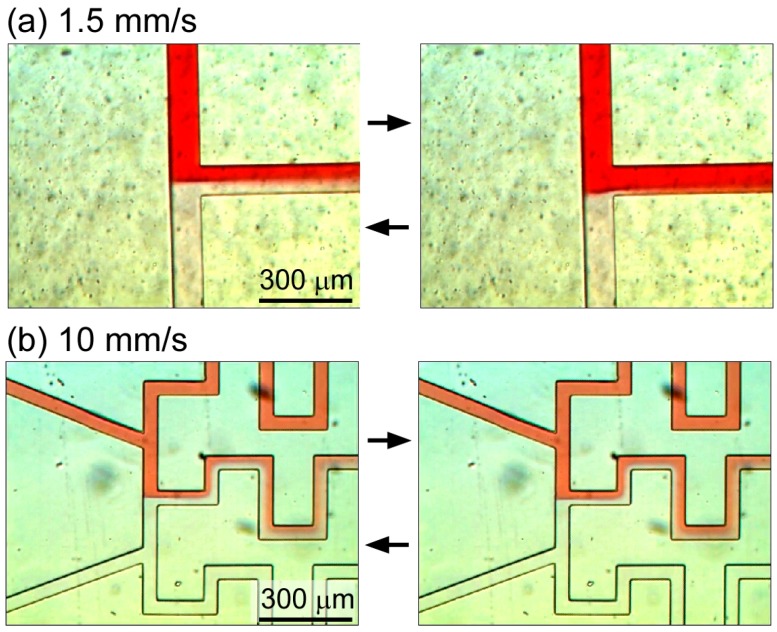
Fluctuation of the interface of the red-dyed and non-dyed solutions at the T-junction of the central microchannel. The interface was moved back and forth as shown in left and right images. (**a**) Fluctuation in the 100 μm × 170 μm microchannel; (**b**) Fluctuation in the 50 μm × 50 μm microchannel.

Our preliminary experiments revealed that high flow velocities in the microchannels are essential to generate a stable concentration gradient. At a velocity of 1.5 mm/s, the position of the interface of the high- and low-concentration glucose solutions inside the microchannel fluctuated by up to 32 μm (Figure 4a), whereas at 100 mm/s, this fluctuation was restricted to 7 μm (Figure 4b), which was small enough to generate a stable concentration gradient. Accordingly, the microchannel dimensions required to generate a flow velocity of 10 mm/s at a flow rate of 1.5 μL/min were determined to be 50 μm (height) × 50 μm (width).

**Figure 5:**
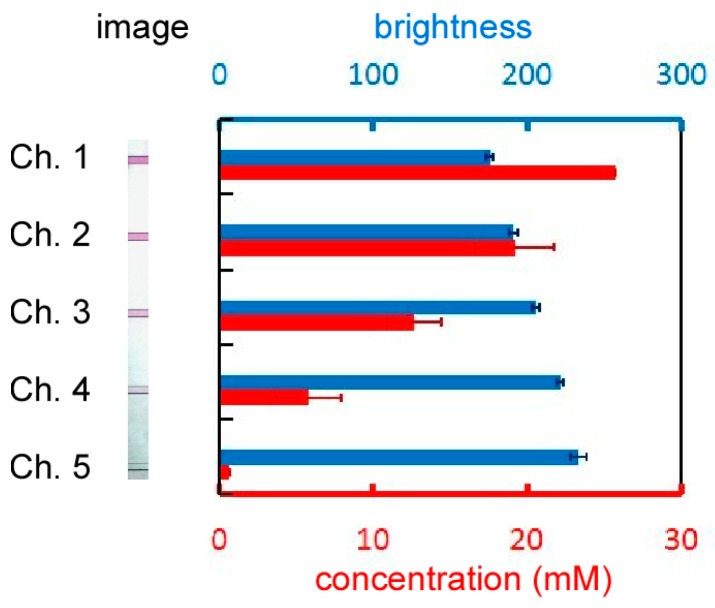
Brightness (blue bars) of the solutions in the microchannels as a function of the concentration (red bars). The diffusion coefficient of Nile red dye is almost the same as that of glucose.

**Figure 6:**
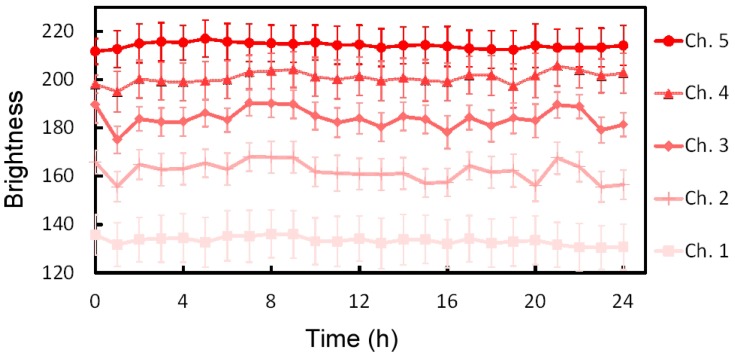
Stability of the brightness of the solutions in the microchannels. The standard deviations of the brightness in all microchannels were less than 3.9.

Solutions with glucose concentration gradients can be obtained when dyed and non-dyed solutions are introduced from the two inlets of the gradient generator (Figure 5). We measured the concentration of the dye (Nile red, Wako Pure Chemical Industry, Osaka, Japan) instead of that of glucose because according to the Einstein–Stokes equation, the diffusion coefficient of the dye in ethanol at 63 °C (7.65 × 10^−10^ m^2^/s) is close to that of glucose in water at 37 °C (7.62 × 10^−10^ m^2^/s):

*D* = *k*_B_*T*/6πη*r* (1)

where *D* is the diffusion coefficient, *k*_B_ is the Boltzmann constant, *T* is the temperature, η is the viscosity, and *r* is the radius of the molecular particle, which is deduced from the molecular weight [30]. Dye concentration is proportional to the brightness of the solution (Figure S1); hence, the relative dye concentrations in the microchannels can be calculated from the brightness of the solution. Solution brightness in microchannels 1–5 was 175.9, 190.8, 205.7, 221.6, and 233.4, respectively. The concentrations of the high- and low-concentration glucose solutions used in the experiment were set at 25.7 and 0.7 mM, respectively. Accordingly, glucose concentrations in microchannels 1–5 were determined to be 25.7, 19.2, 12.7, 5.8, and 0.7 mM, respectively. Furthermore, the stability of the concentrations was analyzed using red-dyed and non-dyed diluted solutions (Figure 6). The standard deviations of the brightness of all solutions over a period of 24 h were less than 3.9, corresponding to 0.7 mM in concentration.

#### 2.1.4. Shear Stress inside the Cell Culture Microchamber

**Figure 7:**
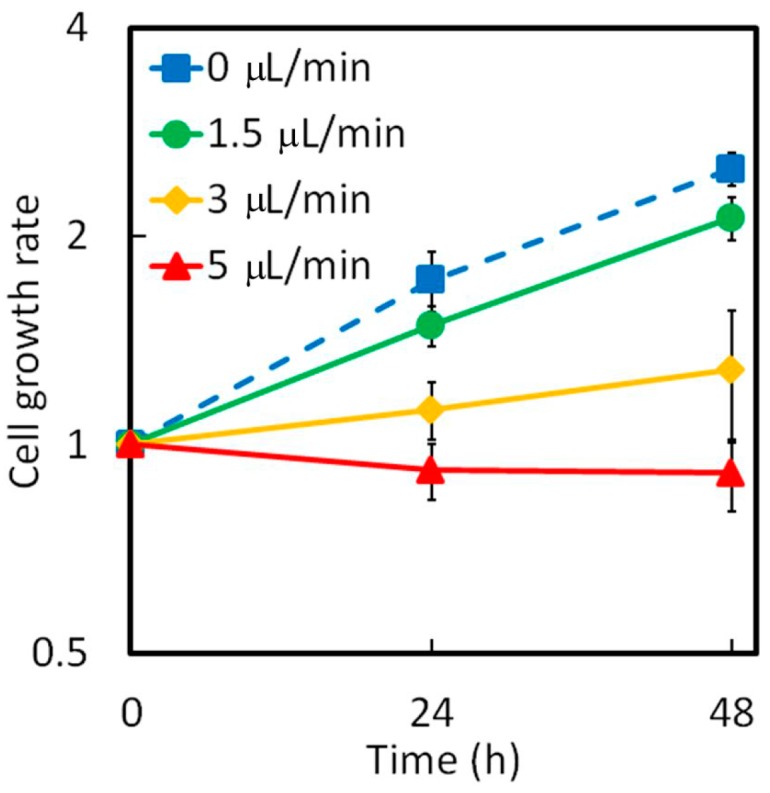
Influence of shear stress in the microchamber on the HeLa cells.

Two types of cell culture microchambers were designed: discrete type (for investigating cellular behaviors in media with uniform glucose concentrations) and united type (for investigating cellular behaviors in media with a glucose concentration gradient). Higher flow velocities in the concentration gradient generator ensure a more stable concentration gradient; however, in the cell culture microchamber, high velocities hamper cell growth because of the shear stress induced on the cells. This shear stress can be calculated using the following equation:

τ = 6μ*Q*/*bh*^2^ (2)

where τ is the shear stress, μ is the viscosity of the solution, *Q* is the flow rate, and *b* and *h* are the width and height of the microchamber, respectively [31]. A shear stress of up to 2.8 mPa, which corresponds to a flow rate of 5 μL/min in our experimental setting (Figure 7), does not affect cellular activities such as proliferation [32]. However, the number of HeLa cells at a flow rate of 5 μL/min decreased with time. At 3 μL/min, the cell growth rate was still appreciably smaller than that in the case without flow. The cell growth rate at 1.5 μL/min was almost the same as that under a shear stress of 0.8 mPa. Furthermore, the flow rate is consistent with the interstitial flow rate on an order of 0.1 μm/s [33], considering the resistance between the channel wall and the solution reduce the velocity to approximately one tenth. Hence, the dimensions of the two microchambers were such that they limited the maximum shear stress acting on the cells to 1 mPa, which is sufficiently low to allow for cell growth.

##### Discrete Cell Culture Microchamber

**Figure 8:**
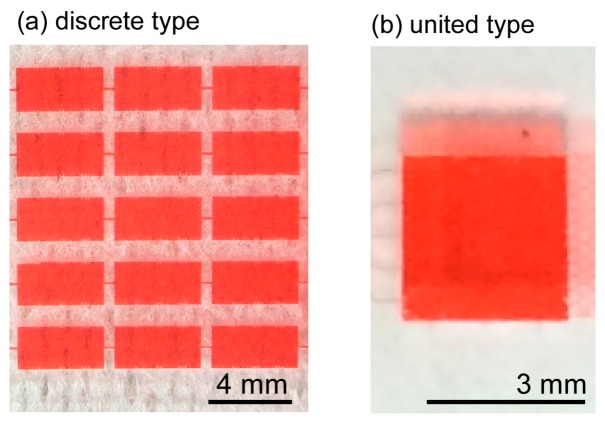
Cell culture microchamber. (**a**) Discrete-type chamber for investigating cell behaviors in glucose solutions with uniform concentration; (**b**) United-type chamber for investigating cell behaviors in glucose solutions with a concentration gradient.

The dimensions of the discrete cell culture microchamber were 2 mm (length) × 4 mm (width) × 170 μm (height) (Figure 8a), which limited the shear stress in the microchamber to 0.68 mPa. Three serially connected microchambers were positioned in parallel.

##### United Cell Culture Microchamber

**Figure 9:**
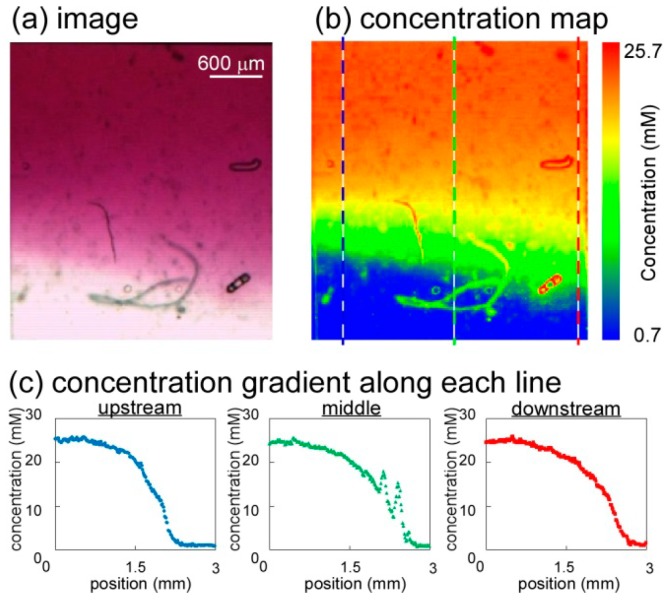
Map of the dye concentration in a united cell culture microchamber. (**a**) Image of the cell culture microchamber during gradient generation by using Nile red dye; (**b**) Map of the concentration of Nile red dye in the microchamber. The diffusion coefficient of the dye is nearly the same as that of glucose, meaning that the concentration of the dye is an accurate proxy for that of the glucose concentration; (**c**) Plot of concentration gradient along each line of upstream, middle and downstream.

**Figure 10:**
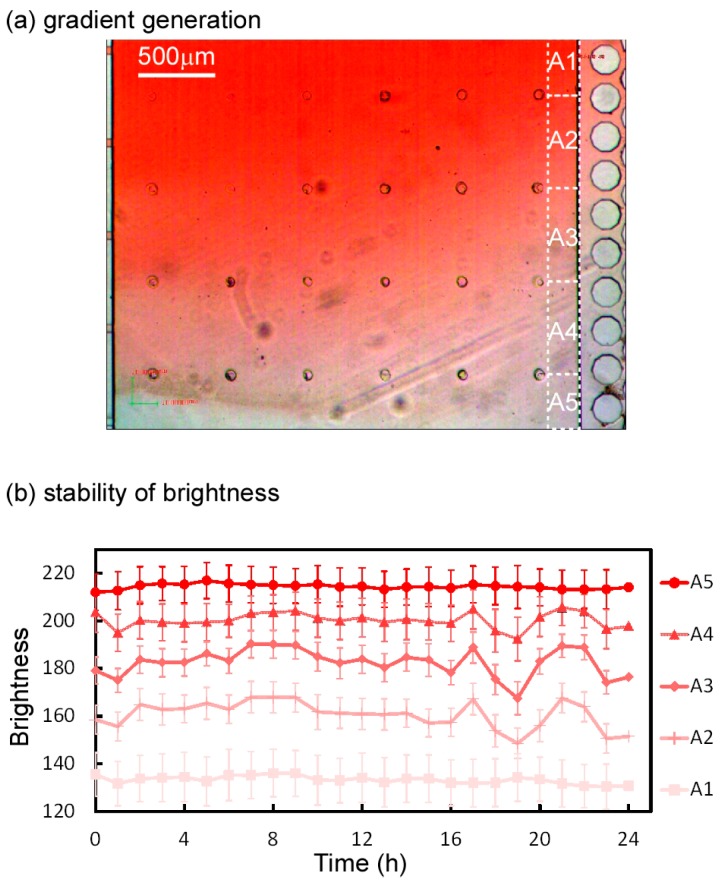
Stability of the brightness of the solution in a united cell culture microchamber. (**a**) Concentration gradation of the red-dyed solution in the cell culture microchamber; (**b**) Stability of the brightness in subsections A1–A5. Standard deviations of the brightness in all subsections were less than 5.7.

The dimensions of the united cell culture microchamber were 3 mm (length) × 3 mm (width) × 480 μm (height) (Figure 8b), which limited the shear stress in the microchamber to 0.23 mPa. The cell culture microchamber was connected to a concentration gradient generator, resulting in the generation of a concentration gradient (Figure 9a); the width of the region over which the glucose concentration changed from low to high was 1800 μm and the resulting slope of the gradient was 1.39 × 10^−2^ mM/μm. Figure 9b,c presents the map of the concentration gradient and the quantitative changes in the gradient from upstream to downstream, respectively. Five solutions with different glucose concentrations were introduced to the cell culture microchamber. Initially, the distribution of the glucose concentration at the upstream edge of the microchamber was steep and crooked, but it gradually became smooth at the middle and downstream edge. The stability of the concentration gradient was analyzed at the downstream section of the united cell culture microchamber by using red-dyed and transparent diluted solution (Figure 10a). The downstream section was divided into five subsections (A1–A5), and the average concentration in each area was plotted (Figure 10b). The standard deviations of the brightness of all solutions over a period of 24 h were less than 5.7, corresponding to 1.0 mM in concentration.

#### 2.1.5. Microfluidic Device to Study Cell Behavior

**Figure 11:**
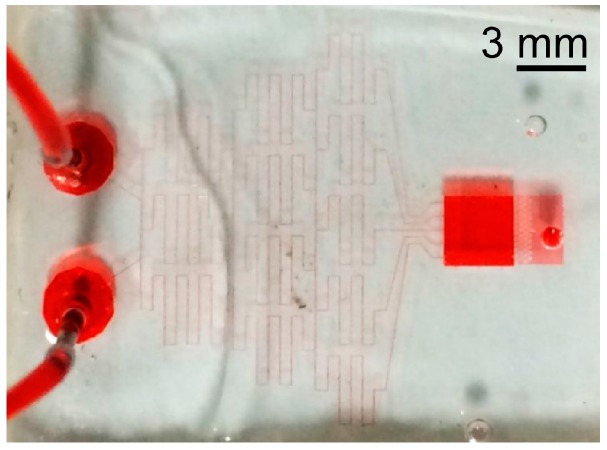
Fabricated microfluidic device to study cell migration inside a tumor.

Figure 11 shows the microfluidic device fabricated to study the cell behavior. The device consists of the microfluidic concentration gradient generator with a small-cross-section and the cell culture microchamber with a large cross-section. It achieves both a stable concentration gradient and a stress-free cell culture environment. Cell behavior in the microchamber can be observed in solutions with different glucose concentrations.

### 2.2. Experimental Setup

**Figure 12:**
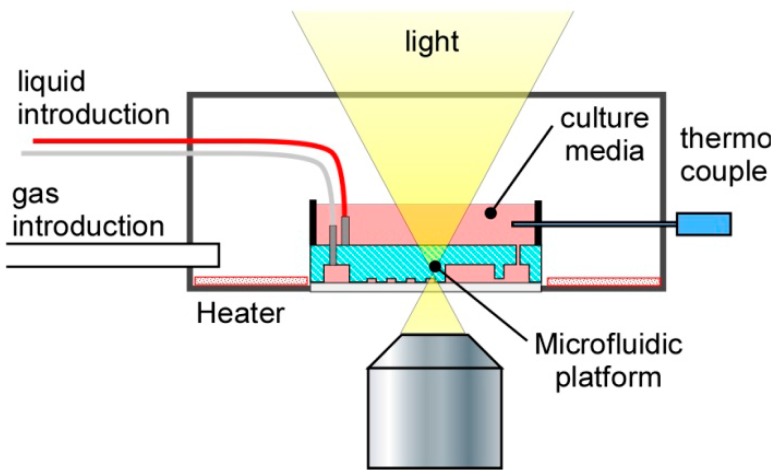
Experimental Setup.

The microfluidic device was placed in a homemade incubation box (Figure 12) in which the gases, solutions, temperature, and humidity could be controlled. A syringe pump (KD-200, KD scientific, Holliston, MA, USA) was connected to the microfluidic device for introducing the solutions. The solution flowed into the microfluidic device at approximately 1.5 μL/min. A gas line (Air:CO_2_ = 100:6) maintained the CO_2_ concentration at 5.2%, which is appropriate for cell culture. Using a heater and a thermocouple, the temperature was maintained at 37 °C. Humidity was maintained at >80% to mimic the conditions inside a typical CO_2_ incubator. Cell behaviors under these conditions were observed using an inverted optical microscope (IX-73, Olympus, Tokyo, Japan). The positions of migrating cells were recorded every hour by time lapse imaging using a charge coupled device (CCD) camera (STC-TC202USB-AS, SENTECH, Kanagawa, Japan), and subsequently relative positions of the cells with respect to steady points in the images were analyzed using a software Tracker (comPADRE, Aptos, CA, USA). This process was performed on all images to obtain the trajectories of cells for 24 h.

#### 2.2.1. Preparation of Cell Suspensions

The HeLa cell line, which were obtained from ATCC (Manassas, VA, USA), is one of the most widely used human cell lines derived from human cervical cancer cells. HeLa cells were cultured with Dulbecco’s Modified Eagle Medium (DMEM, D-MEM with L-glutamine, phenol red, and sodium pyruvate; Wako Pure Chemical Industry, Osaka, Japan) containing fetal bovine serum (FBS, regular; Corning) inactivated at 56 °C for 30 min, penicillin, and streptomycin (PS, Penicillin-Streptomycin solution 100X, Wako) in the following proportion: 10% FBS, 100 unit/mL penicillin, and 100 mg/mL streptomycin. We prepared DMEM containing a high glucose concentration (25 mM) and no glucose, and the FBS contained 7.2 mM of glucose. Therefore, the final glucose concentrations of the high- and low-concentration glucose media were 25.7 and 0.7 mM, respectively. The cells were maintained in the culture medium containing high glucose (25.7 mM). The doubling time of the HeLa cells cultured with the medium containing high glucose was almost 1 day at 37 °C, 5% CO_2_ concentration, and sufficiently high humidity. After sufficient cultivation, the HeLa cells were retrieved using trypsin solution, collected by centrifugation, and diluted using DMEM, resulting in a suspension with a cell density of 1 × 10^5^ to 1 × 10^6^ cells/mL.

#### 2.2.2. Experimental Conditions

**Figure 13:**
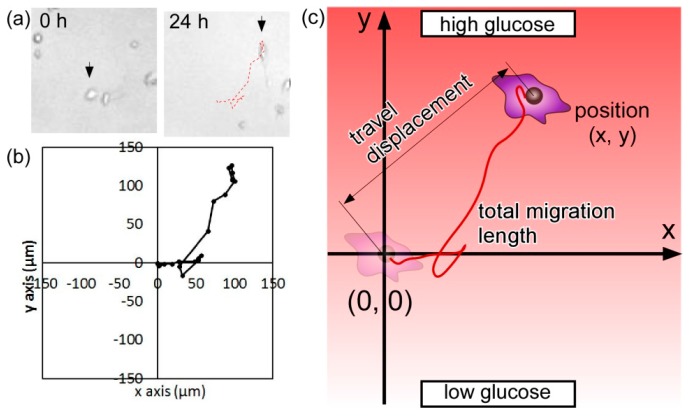
Parameters used to analyze the mobility of HeLa cells. (**a**) Images of HeLa cells at the beginning and 24 h after the experiment. Red dashed line indicates the trajectory of a cell; (**b**) Cell trajectory over a period of 24 h; (**c**) Parameters used to describe cell mobility.

The microfluidic device was sterilized at 127 °C and 500 kPa for 30 min using an autoclave. A collagen solution (Cellmatrix Type IC, Nitta Gelatin, Osaka, Japan), diluted with dilute hydrochloric acid (HCl) in the ratio 1/10, was introduced into the microchamber at 5 μL/min and was dried on the clean bench for 12 h to cover the surface of the microchamber with the collagen. The microchannels and the microchamber were filled with phosphate buffer solution (PBS, Wako) and exposed to ultraviolet (UV) light for 30 min. Subsequently, the channels and the chamber were washed with PBS at 5 μL/min until the extra collagen and contaminants were removed. The microfluidic device was filled with the high glucose DMEM at 10 μL/min to sufficiently wash the microchambers. The prepared cell suspension was then introduced into the microchambers. The microfluidic device was placed inside the incubation box maintained at 37 °C, 5% CO_2_ concentration, and sufficiently high humidity. The cells inside the microfluidic device had strong adhesion in the high glucose medium 12 h after cell introduction. The high- and low-concentration glucose DMEMs were introduced from the two inlets at 1.5 μL/min to generate the glucose concentration gradient inside the microchambers. To analyze cell migration and mobility of the HeLa cells, the initial (origin) and final positions of the HeLa cells were traced under each condition over a period of 24 h (Figure 13a,b) in order to determine their travel distance (i.e., distance between the origin and the final position) and total migration length (i.e., length of trajectory of cell migration) (Figure 13c). Furthermore, we introduced indicators to describe the directionality (i.e., ratio of net cell displacement to total migration length) and chemotactic index (CI, i.e., ratio of travel displacement toward the gradient and total migration length). For the ease of analysis of the cell migration and mobility, we focus on the cells without cell division.

## 3. Results

### 3.1. Growth Rate and Motion of HeLa Cells under Different Glucose Concentrations

**Figure 14:**
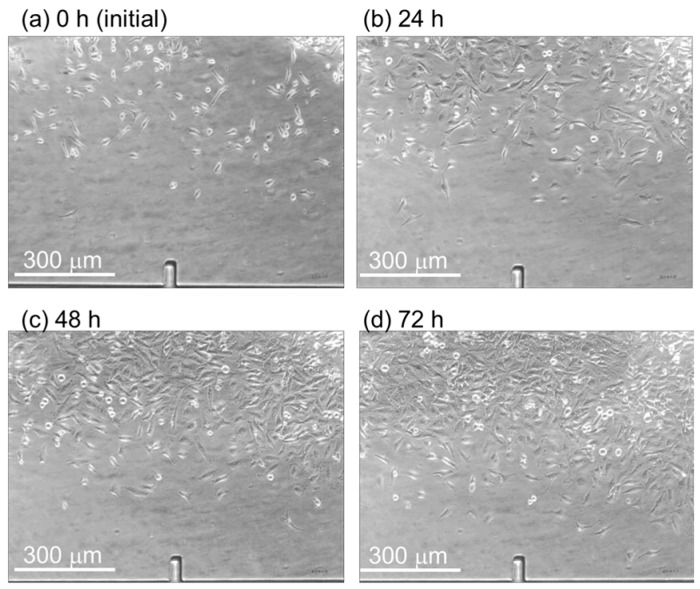
Cell proliferation in the cell culture chamber. The HeLa cells grew from (**a**) the initial state to (**b**) 24 h; (**c**) 48 h; and (**d**) 72 h.

**Figure 15:**
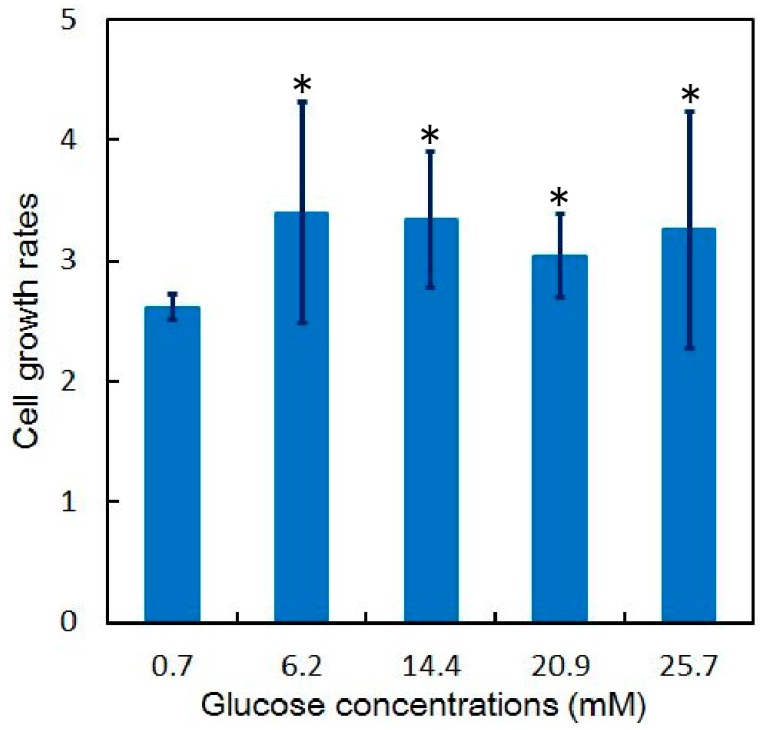
Growth rate of HeLa cells in discrete cell culture microchambers (*n* = 3); * *p* < 0.05 (*t*-test) in 0.7 mM.

By using the microfluidic device with the discrete cell culture microchamber, HeLa cells were introduced into the separated microchambers and cultured by perfusing DMEMs of different glucose concentrations: 25.7, 19.2, 12.7, 5.8, and 0.7 mM. The flow rate in the microchamber was 1.5 μL/min, and the corresponding applied shear stress was 0.23 mPa. The HeLa cells were cultured in a solution with uniform glucose concentration for 72 h. The HeLa cells divided and the number increased from that at the initial state (Figure 14a), to those at 24 h (Figure 14b), 48 h (Figure 14c), and 72 h (Figure 14d). At concentrations of 5.8–25.7 mM, the number of the HeLa cells cultured increased 3.0- to 3.4-fold (*n* > 200 at the initial condition under each glucose concentration) relative to the initial number, whereas at 0.7 mM, the number of cells increased by a factor of 2.6 (*n* = 520 at the initial condition) (Figure 15).

**Figure 16:**
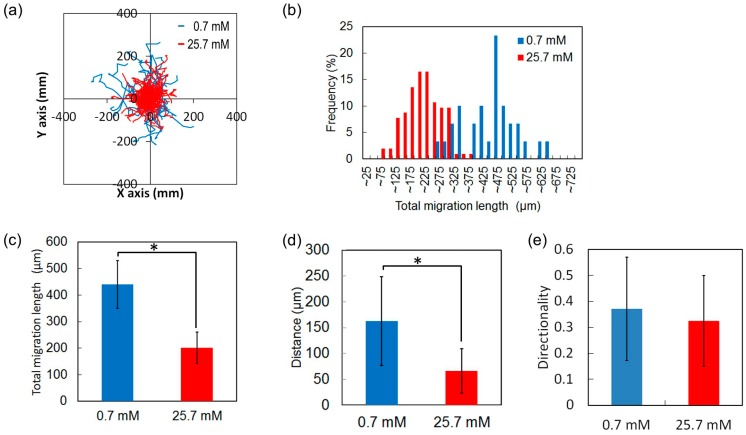
Mobility of HeLa cells at low and high glucose concentrations over a period of 24 h. (**a**) Trajectories of HeLa cells; (**b**) Histogram of the total migration length; (**c**) Average total migration length; (**d**) Travel distance from the origin; (**e**) Directionality along the y-axis. * *p* < 0.01 (*t*-test).

The mobility of the HeLa cells grown in 0.7 (*n* = 30) and 25.7 mM (*n* = 103) glucose measured over a period of 24 h exhibited different growth rates. The cells were uniformly distributed. According to the trajectories of all cells and the histogram of the total migration distance at each concentration, HeLa cells cultured in 0.7 mM glucose randomly travelled farther than did those in 25.7 mM glucose (Figure 16a,b). The total migration lengths of HeLa cells cultured in 0.7 and 25.7 mM glucose were 441 and 201 μm, respectively (Figure 16c). The deviation and distance from the origin of cells cultured in 0.7 mM glucose were larger than those cultured in 25.7 mM glucose (Figure 16d), assuming that the origin of all cells was the same. The average distance covered by the HeLa cells cultured in 0.7 and 25.7 mM glucose was 162 and 66 μm, respectively. The directionalities of the two groups of HeLa cells did not differ (Figure 16e). These results suggest that a low glucose concentration reduces the growth rate but increases the mobility of the HeLa cells. This may be because the deprivation of glucose causes the regulation of proliferation of the cells, because glucose serves as a precursor for DNA synthesis and ATP generation [34].

### 3.2. Motion of HeLa Cells under Uniform and Gradient Low Glucose Concentrations

**Figure 17:**
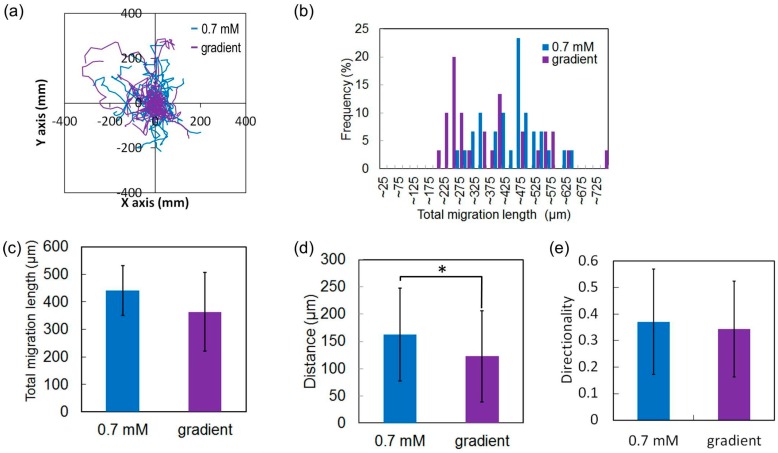
Mobility of HeLa cells in uniform and low gradient concentrations of glucose over a period of 24 h. (**a**) Trajectories of HeLa cells; (**b**) Histogram of the total migration length; (**c**) Average total migration length; (**d**) Travel distance from the origin; (**e**) Directionality of HeLa cells along the *y*-axis. * *p* < 0.01 (*t*-test).

**Figure 18:**
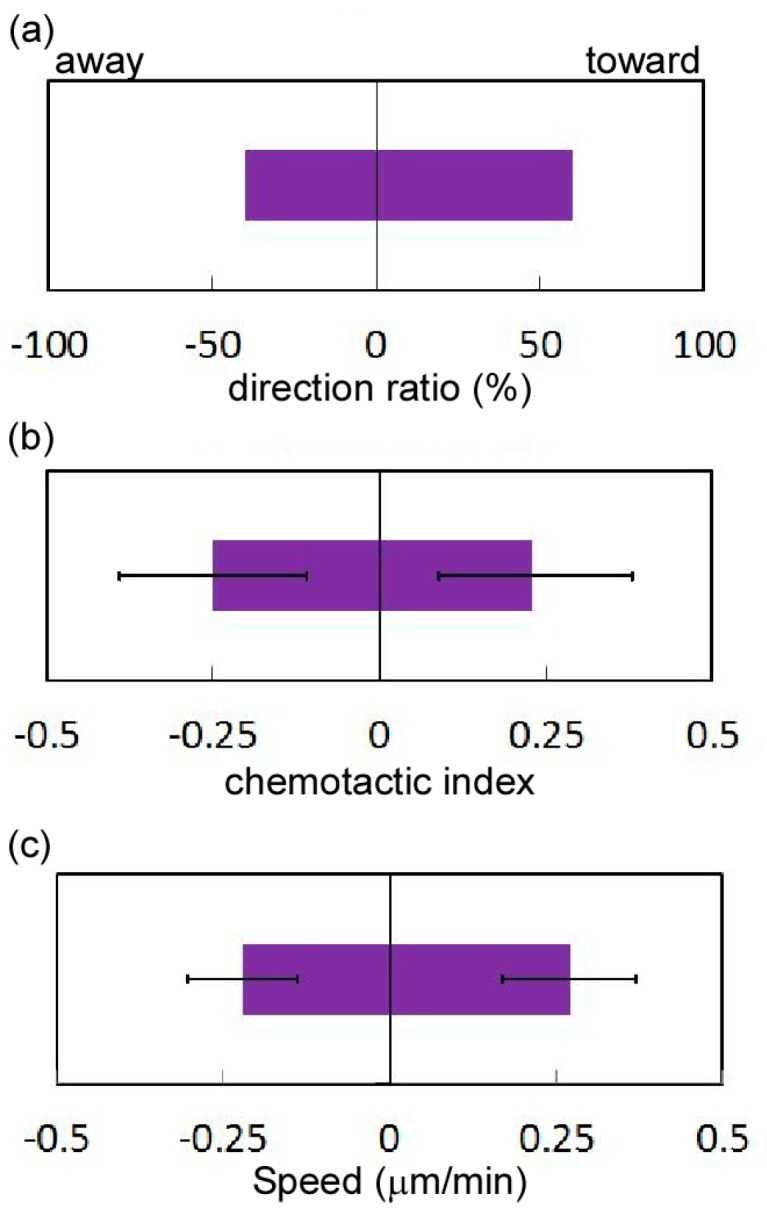
Analysis of the chemotaxis of HeLa cells. (**a**) Direction ratio; (**b**) Chemotactic index; (**c**) Migration speed toward and away from the high-concentration region.

The aforementioned results reveal the differences in the migration behavior of HeLa cells grown in glucose solutions with high and low concentrations. On the basis of these results, we hypothesize that HeLa cells migrate from regions of low glucose concentration to those of high concentrations. To test the hypothesis, we compared the behaviors of 30 HeLa cells grown in 0.7 mM glucose concentration and in a glucose solution with a concentration gradient of 1.6 to 7.9 mM by using the microfluidic device with the united cell culture microchamber over a period of 24 h. The trajectories and the histogram of the total migration distance of both groups of cells indicated that both groups of cells moved randomly (Figure 17a). Total migration lengths of the two groups differed non-significantly (Figure 17b), but the average total migration distances were similar (441 and 364 μm; Figure 17c). The positions of both groups of cells 24 h after the experiment began were randomly distributed (Figure 17a), indicating the lack of directional migration in both investigated cases. The deviations and distances from the origins in both cases were almost the same (Figure 17d,e). The average travel distance of the HeLa cells grown in the 0.7 mM glucose concentration and in the low glucose range (1.6–4.8 mM) was 162 and 122 μm, respectively (Figure 17d), whereas the corresponding directionalities did not differ (Figure 17e). Furthermore, we analyzed in detail the chemotactic migration of HeLa cells under the glucose gradient in terms of the direction ratio, CI, and speed. HeLa cells were classified into two groups on the basis of their directionality: those moving toward the high-concentration region (i.e., cells with positive CIs) and those moving away from the high-concentration region (i.e., cells with negative CIs); 60% and 40% of the HeLa cells had positive and negative CIs, respectively (Figure 18a). The CI of all HeLa cells was 0.040, whereas that for cells that moved toward and away from the high-concentration region were 0.23 and −0.25, respectively (Figure 18b). The migration speeds of the cells with positive and negative CIs were 0.27 and 0.22 μm/min, respectively (Figure 18c), which were quite close to the speeds of HeLa cells grown under low glucose concentrations. Additional experiments showed that HeLa cells randomly migrated even in glucose solutions with concentration gradients that were twice as steep in the experiment, where the width of the transition from low (0.7 mM) to high (25.7 mM) concentration was 900 μm, resulting in a concentration gradient of 2.78 × 10^−2^ mM/μm, which is comparable to the glucose concentrations in the human body. These results suggest that HeLa cells grown in a glucose solution with a concentration gradient exhibit random motion rather than chemotaxis. We need further study of cell migration responding to glucose because a mechanism of glucose-induced cellular mobility seems to depend on cell types [35,36].

## 4. Conclusions

We developed a microfluidic device with narrow channels and a wide cell culture chamber to achieve a favorable balance between the stability of the glucose gradient and the shear stress on the cells and studied the behavior of cells grown in different glucose concentrations. HeLa cells exhibited increased mobility at low glucose concentrations and maintained random motion even in solutions with a glucose concentration gradient. The developed device can precisely modify the chemical conditions and is valuable in studying the chemotaxis response of cancer cells to different glucose concentrations. Through a combination of global analysis of genes and proteins related to the cell behaviors reported in this paper, we may be able to reveal the regulatory mechanisms of cancer cell migration, leading to a more comprehensive understanding of cancer metastasis.

## Supplementary Materials

The following supplementary materials are available online at www.mdpi.com/2072-666X/7/9/155/s1, Figure S1: Calibration between dye concentration and brightness.
