# Supplementary material for: Investigation of the Influence of Glucose Concentration on Cancer Cells by Using a Microfluidic Gradient Generator without the Induction of Large Shear Stress
Source: Micromachines (Basel). 2016 Sep 1;7(9):155. doi: 10.3390/mi7090155 (PMC6189924; doi:10.3390/mi7090155)
Supplement: Supplementary file 1 [file micromachines-07-00155-s001.pdf]

Supplementary Materials: Investigation of the Influence of Glucose Concentration on Cancer Cells by Using a Microfluidic Gradient Generator without the Induction of Large Shear Stress

Tadashi Ishida, Takuya Shimamoto, Nobuya Ozaki, Satoshi Takaki, Takahiro Kuchimaru, Sinae Kizaka-Kondoh and Toru Omata

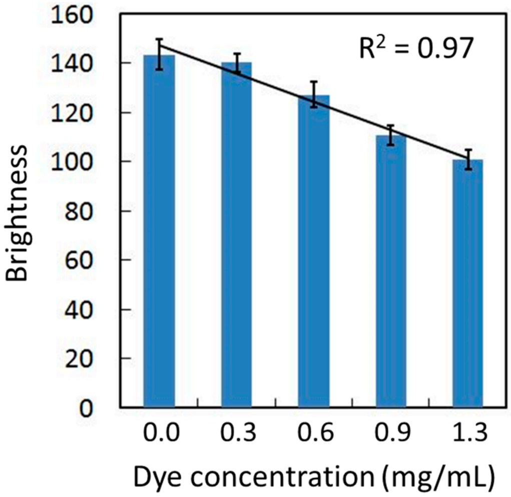

Figure S1. Calibration between dye concentration and brightness. The brightness of the colored water using the dye is linear in the range of our usage. The R-squared value is 0.97 when the data is linearly fitted.
